# Placental Mesenchymal Dysplasia With Normal Placental Growth Factor Levels Complicated by Fetal Giant Liver Cyst and Anemia

**DOI:** 10.7759/cureus.81377

**Published:** 2025-03-28

**Authors:** Yoo E An, Elisabeth Smet

**Affiliations:** 1 Obstetrics and Gynecology, Westmead Hospital, Sydney, AUS; 2 Maternal and Fetal Medicine, Westmead Hospital, Sydney, AUS

**Keywords:** foetal anaemia, foetal liver cyst, placental growth factor, placental mesenchymal dysplasia, placentomegaly

## Abstract

A case is presented of a 27-year-old woman who was referred to our Maternal Fetal Medicine Center following the detection of a multi-cystic placenta on a routine morphology scan. Ultrasound at our unit confirmed the presence of a diffusely cystic placenta and a well-grown and structurally normal fetus. The patient's placental growth factor (PlGF) level was found to be within normal limits. At 23 weeks, an enlarging hepatic cyst was identified in the fetus and was monitored through serial imaging. A multidisciplinary team was involved, including neonatal intensivists and pediatric surgeons. The patient underwent an emergency cesarean section at 30+4 weeks of gestation after presenting with preterm labor and concerns of fetal deterioration. She delivered a live-born female infant of normal birth weight. However, the neonate required immediate resuscitation with multiple blood product transfusions, likely due to complications from the known giant hepatic cysts. She was transferred to a quaternary neonatal unit for further management. Genetic testing excluded Beckwith-Wiedemann syndrome, which had been an antenatal differential. The neonate was discharged 2.5 months later after multiple drainages of her cysts, which were benign on fluid cytology. Our case highlights the complexity of placental mesenchymal dysplasia (PMD) and the importance of early multidisciplinary care to achieve the best possible outcome. The study also underlines the need for further research into the prognostic reliability of PlGF levels in PMD.

## Introduction

First described in 1991, placental mesenchymal dysplasia (PMD), also known as "pseudo hydatidiform mole," is an uncommon diagnosis with an estimated worldwide prevalence of 0.02% of all pregnancies. The condition is characterized by placentomegaly, vascular abnormalities, stem villous cystic dilation with vesicle formation of the placenta, and has a high female predominance [1]. It has been associated with diverse fetal outcomes, from uncomplicated pregnancies to intrauterine demise and maternal complications, especially pre-eclampsia [1-2]. By 2022, just over 100 cases of PMD had been reported in the literature, and its rarity makes it an uncommon diagnosis in the field of obstetrics [3]. It is essential to consider PMD in the diagnosis of cystic placental lesions. Differentiating PMD from other conditions, such as a partial molar pregnancy with an abnormal triploid fetus, is critical as the latter may lead to pregnancy termination [4]. Definitive diagnosis of PMD is through histopathology, which consists of mesenchymal hyperplasia, edema of stem-cell villi, dilated stem vessels with thickened vasculature, and absence of trophoblastic hyperplasia [5]. Immunohistochemistry for p57 in PMD demonstrates staining of villous cytotrophoblast cells and a loss of staining in stromal cells of dysplastic stem villi [1]. Early identification and diagnosis will allow appropriate counselling on the associated risks, which include pre-eclampsia, fetal and neonatal death, and the possibility of Beckwith-Wiedemann syndrome (BWS) [1]. While placental growth factor (PlGF) has been proposed as a prognostic marker of placental function [6], its role in PMD remains unclear due to limited data on the incidence and complications of this condition. Further research is essential to improve management strategies, including investigations, delivery timing, and patient counseling.

## Case presentation

Antenatal

**Figure 1:**
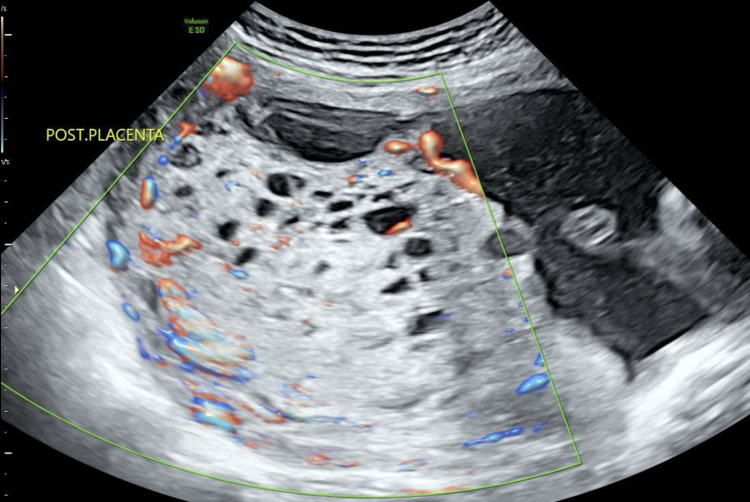
Ultrasonography at 19+4 weeks confirming a diffusely cystic appearance of the placenta with "stained glass sign"

A 27-year-old G3P2 was referred to the maternal fetal medicine department for review of a cystic placenta noted on a routine morphology scan. Her history included two previous cesarean deliveries (x1 for fetal distress, x1 elective repeat) of two healthy neonates with normal birth weights. She had no significant personal medical or family history of note. Aneuploidy screening was declined. Ultrasound confirmed a diffusely cystic placenta with moderate vascularity within (stained glass sign) (Figure 1) [7].

The absence of hyper-reaction luteinalis, combined with a structurally normal and well-grown fetus, favored the diagnosis of PMD. Elevated serum alpha-fetoprotein levels and beta-human chorionic gonadotropin levels supported this diagnosis. Differentials included a molar pregnancy, chorioangioma, and subchorionic hematoma [8]. However, her PlGF levels were within normal limits, suggesting adequate placental function (Table 1) [5].

**Table 1:** Laboratory results of the patient

Variables	Patient results	Reference range
Alpha-foetoprotein (AFP)	151 kIU/L	≤6 kIU/L
Beta-human chorionic gonadotropin (bHCG)	103,084 IU/L	≤1 IU/L
Placental growth factor (PlGF)	164 pg/mL	<100 pg/mL

**Figure 2:**
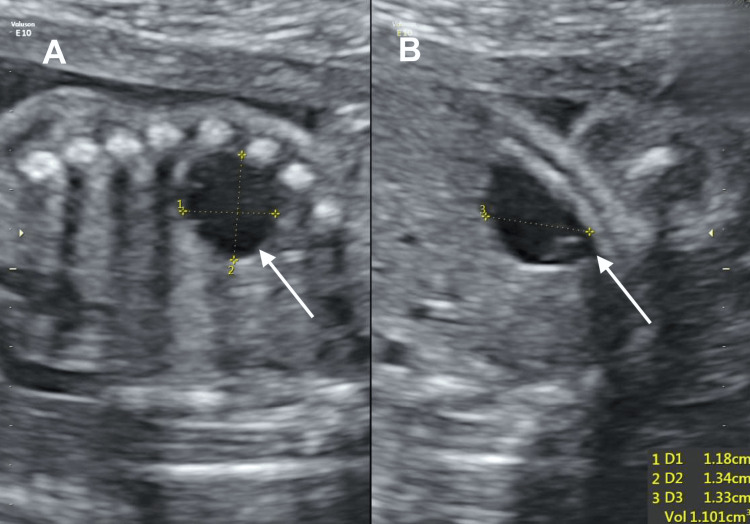
Ultrasonography at 23+4 weeks showing an avascular hepatic cyst The arrows point to the fetal hepatic cyst seen on ultrasonography. A) Ultrasound showing the presence of an avascular hepatic cyst measuring 1.18 cm x 1.34 cm (D1 = depth, D2 = width) B) Ultrasound showing the presence of an avascular hepatic cyst measuring 1.33 cm (D3 = length)

**Figure 3:**
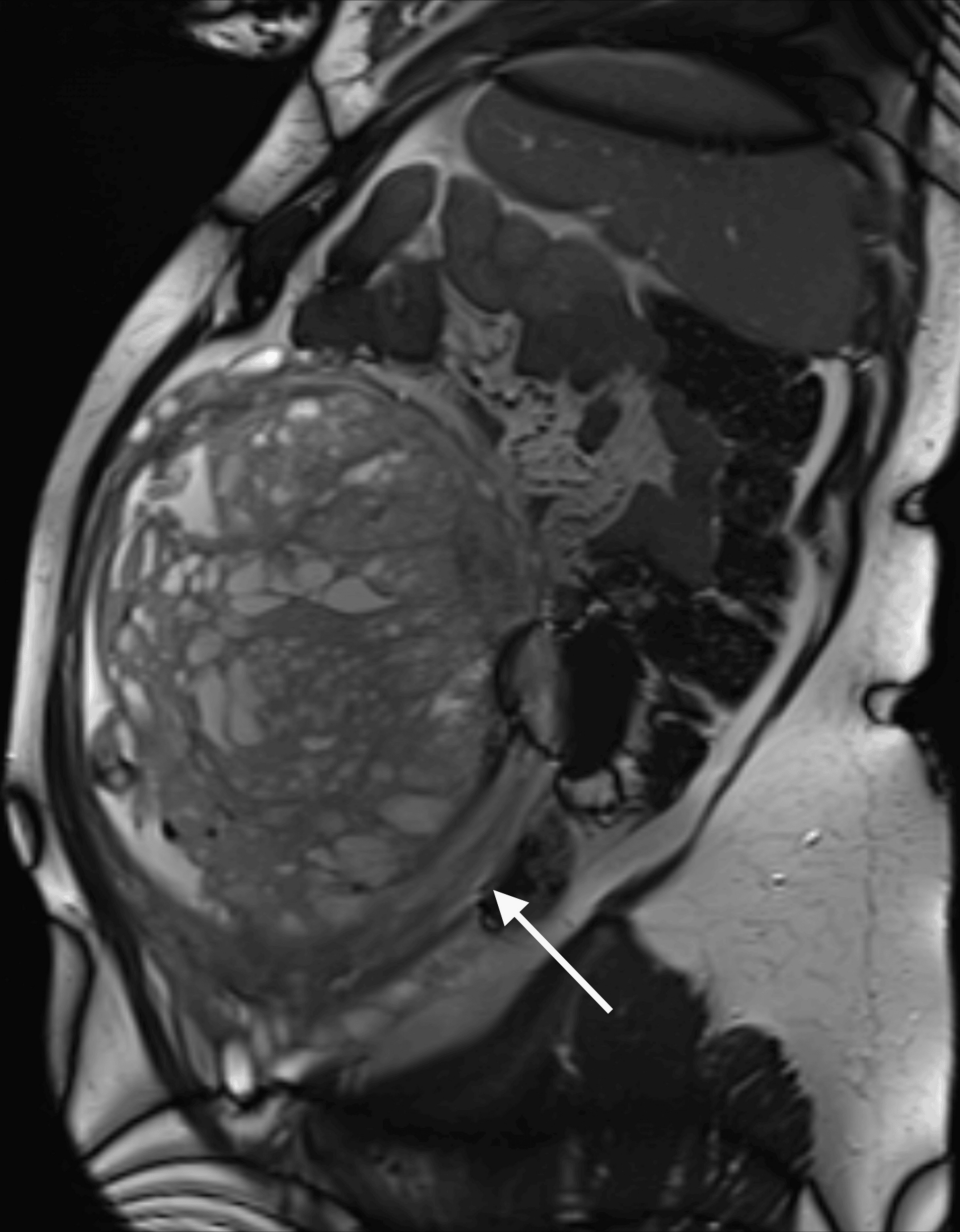
MRI at 28+0 weeks confirming placentomegaly with cystic lesions associated with placental mesenchymal dysplasia The arrow points to the cystic placenta seen on the MRI

**Figure 4:**
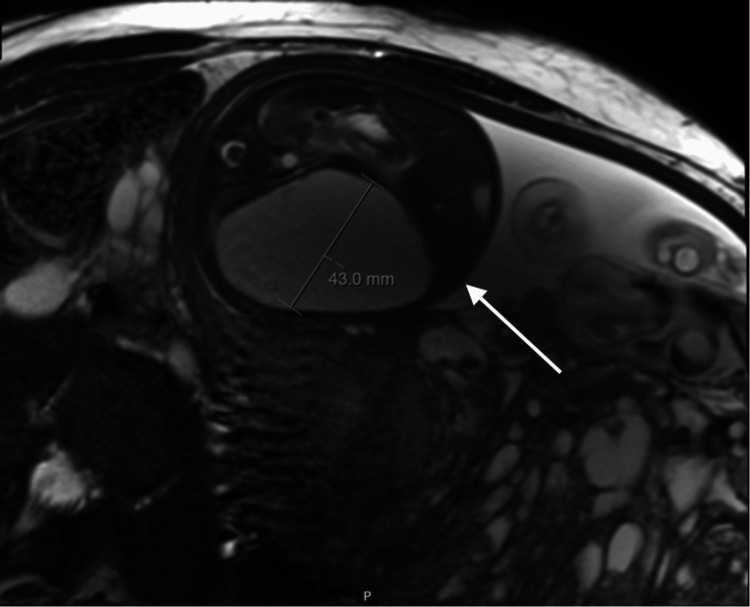
MRI at 28+0 weeks demonstrating an enlarging fetal liver cyst The arrow points to the fetal hepatic cyst seen on MRI

Serial ultrasounds showed normal fetal growth. From 23 weeks onwards, a rapidly enlarging hepatic cyst was identified (Figure 2). BWS was considered, but the patient declined invasive testing. A fetal MRI at 28 weeks confirmed the ultrasound findings (Figures 3-4).

At 30+4 weeks' gestation, the patient presented with reduced fetal movements and signs of early labor. An abnormal cardiotocography (CTG) and elevated middle cerebral artery peak systolic velocity suggested fetal anemia. An emergency cesarean section was required, following a single injection of betamethasone (one hour before surgery) and intravenous magnesium sulfate, for fetal lung maturation and neuroprotection, respectively.

Postnatal

**Figure 5:**
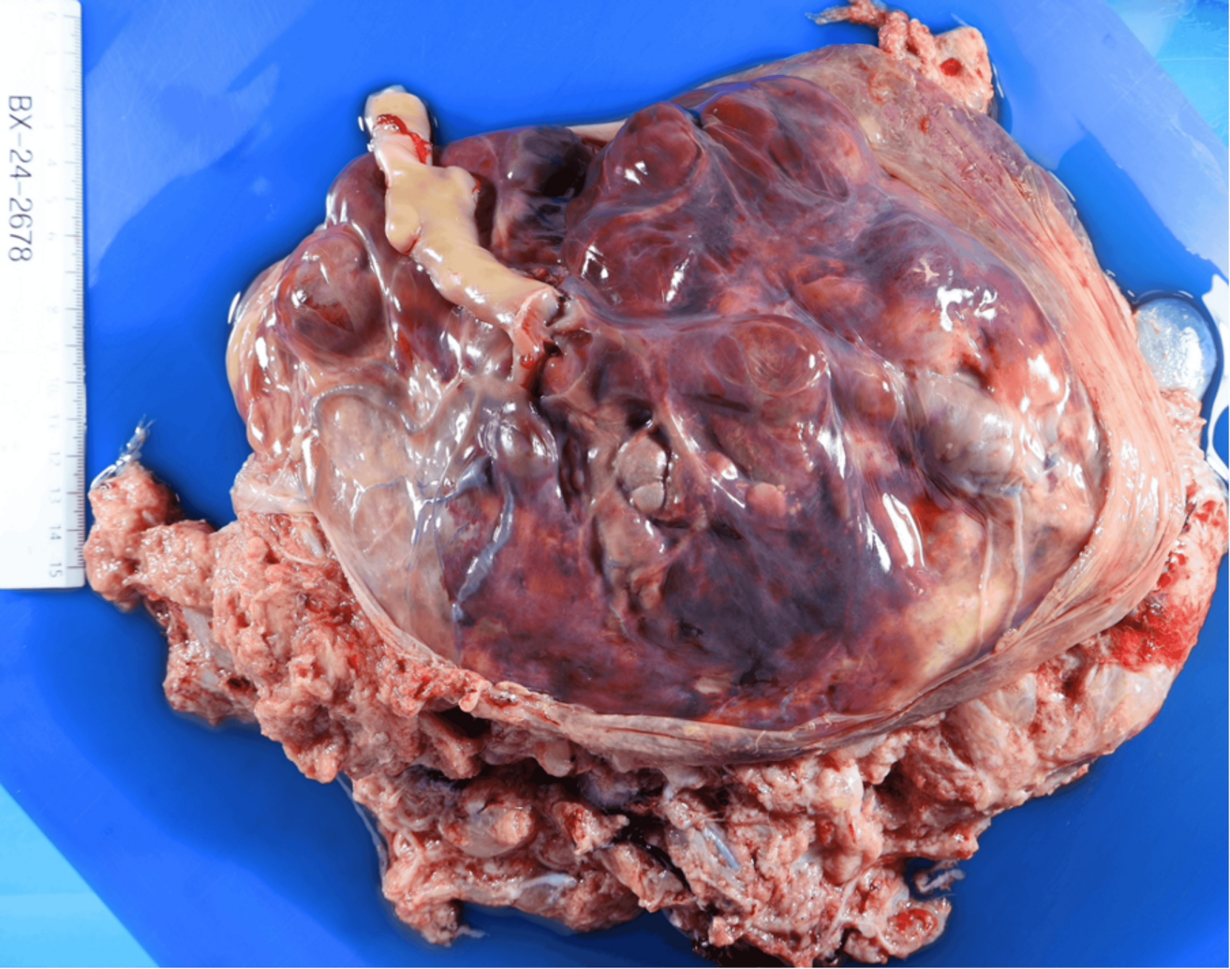
Macroscopic view of placenta

The patient delivered a live-born female with APGAR scores of 6, 6, and 8, and the newborn required immediate resuscitation. There was meconium-stained amniotic fluid, but no signs of placental abruption. The birth weight was 1,513 g (70% percentile), with a placental weight of 1,599 g (>95% percentile; Figure 5). The patient was discharged three days post-delivery with no complications, and her newborn remained in the neonatal intensive care unit (NICU) before being discharged home at 2.5 months.

**Figure 6:**
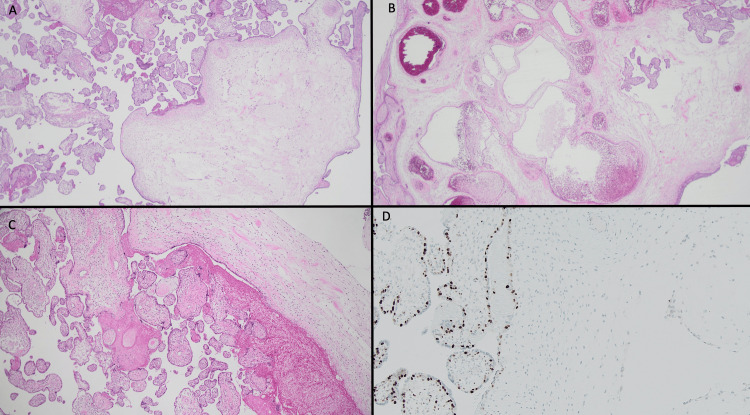
Placenta histopathology and immunohistochemistry A) Placenta histopathology showing cistern formation of stem villi with adjacent normal villi B) Placental histopathology showing dilated and abnormal stem villous vessels C) Placental histopathology showing the absence of trophoblastic hyperplasia D) Placental immunohistochemistry for p57 which is negative in the stromal cells but positive in the trophoblastic component

PMD was confirmed on histopathology and immunohistochemistry (Figure 6).

Newborn

The newborn at delivery had anemia, thrombocytopenia, and prolonged prothrombin time and activated partial thromboplastin time. Her liver function tests were largely normal. She required blood products to treat her pancytopenia (Table 2).

**Table 2:** Laboratory values of the newborn at delivery

Variables	Newborn results	Reference ranges
Hemoglobin	70 g/L	142-240 g/L
Platelet	50x10^9/L	150-400x10^9/L
Prothrombin time	16 seconds	9-13 seconds
Activated partial thromboplastin time	49 seconds	25-37 seconds
Alanine transaminase	31 U/L	7-36 U/L
Aspartate aminotransferase	168 U/L	39-109 U/L
Gamma-glutamyl transferase	91 U/L	33-211 U/L
Alkaline phosphatase	59 U/L	80-380 U/L

**Figure 7:**
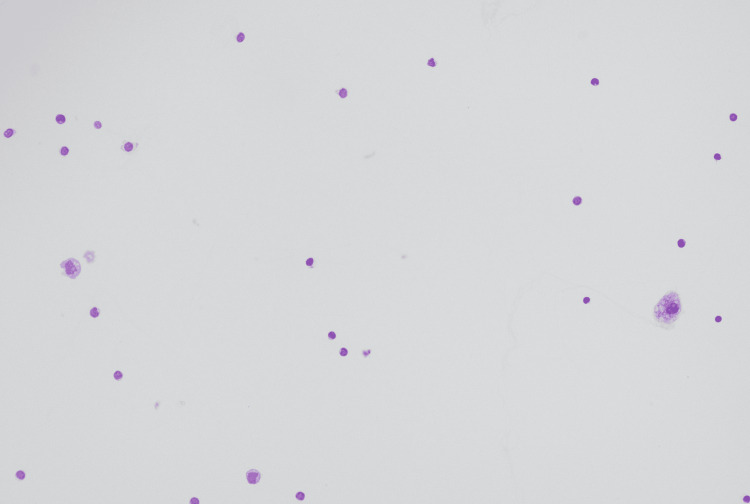
Microscopic view of fluid cytology from the drained liver cyst with no malignant cells Diff-quick stained, x200 magnification of fluid cytology

Due to the known antenatal diagnosis of hepatic cyst causing mass effect, the newborn was transferred to a quaternary NICU for further management. She had two hepatic cysts, one massive multi-septate cyst in the right hepatic lobe and a second small one in the left lobe. The larger cyst occupied almost the entire abdomen, causing abdominal compartment syndrome, resulting in renal failure and significant respiratory compromise. Urgent ultrasound-guided drainage of the cyst was performed, and a drain was left in the cyst with overall improvement. Fluid cytology was benign (Figure 7).

Once the infant was weaned off ventilation, the drain was removed, but the cyst re-accumulated and required a further drain. Despite these interventions, the baby grew appropriately and was discharged home 10 weeks later. The cyst continued to gradually re-accumulate after discharge home, and currently, surgical exploration with biopsy and cyst excision/marsupialization is being considered. BWS was suspected but ruled out by genetic testing.

## Discussion

PMD has been associated with fetal complications, including intrauterine growth restriction and intrauterine fetal death, and maternal complications such as pre-eclampsia [8]. Its association with BWS and fetal hepatic mesenchymal tumors suggests a shared pathogenetic mechanism [9-10].

A 2013 review of PMD cases with fetal liver cysts reported a poorer prognosis when compared to cases without. Nearly half of the case reports of PMD with fetal liver cysts resulted in fetal demise [11]. Our case reinforces this negative association, with the newborn presenting with pancytopenia requiring long-term NICU admission and surgical management of the hepatic cysts. Notably, the feto-maternal hemorrhage quantification was negative, and there was no overt evidence of placental abruption at delivery, which created uncertainty about the underlying etiology of the anemia. It is currently hypothesized that hepatic cyst-induced coagulation disturbances may have contributed to the pancytopenia. Other potential explanations include microangiopathic hemolysis within the abnormal placental vasculature, a disproportion between a normally grown fetus and marked placentomegaly no longer sustained by fetal erythropoiesis, or rupture of the aneurysmal placental vessels.

PlGF, a pro-angiogenic factor essential for placental vascularization and trophoblastic growth, provides a valuable assessment of placental function [12]. Low levels of PlGF have been associated with adverse outcomes, such as preterm birth, early-onset pre-eclampsia, and stillbirth [13]. Therefore, in clinical practice, a normal PlGF level is generally considered to indicate adequate placental function. However, the predictive utility of PlGF in the context of PMD remains inconsistent.

In 2016, two case reports hypothesized the measurement of PlGF levels as a prognostic marker for those with PMD. One of the cases involved a healthy 37-year-old woman with PMD and normal PlGF levels. This case resulted in vaginal delivery of a live-born female at term. The second case was a healthy 30-year-old woman with PMD but low PlGF levels. This case resulted in an emergency C-section at 31+3 weeks due to fetal and maternal deterioration [14].

In our patient, normal PlGF levels correlated with normal fetal growth. While this indicates that PlGF is a helpful test for prognosis in the setting of PMD, this finding does not align with an unpublished PMD case treated at our unit. In that instance, the PlGF level was high-normal despite the fetus being severely growth-restricted, with subsequent demise at 28 weeks of gestation. This case further challenges PIGF’s reliability in PMD prognosis and highlights the need for further research in this setting. We hypothesize that although PlGF is primarily secreted by the syncytiotrophoblast, the mesenchymal overgrowth in PMD may add to the normal PlGF values. While robust evidence for this hypothesis is lacking, a relationship between PlGF and mesenchymal progenitors has been described, although its direct placental mesenchymal secretion remains speculative [15]. If this hypothesis proves correct, PlGF levels are likely not a useful test in cases of PMD.

As a word of caution, in the setting of an abnormal CTG, we would not recommend delaying delivery to perform an ultrasound. While this was not best practice, it allowed us to prepare our neonatologists for expected neonatal anemia and to have blood products readily available in the theater.

Lastly, as our paper is from an obstetric perspective, there are limitations in providing comprehensive details through this case report of the neonate's trajectory from birth to discharge.

## Conclusions

This report underscores the complexity of PMD and the necessity for close monitoring in tertiary centers with early pediatric involvement. Although uncommon, PMD should be a differential diagnosis for ultrasound findings of a cystic placenta with an otherwise structurally normal fetus. Early diagnosis will allow for adequate counseling of the associated maternal and fetal risks (fetal demise, intrauterine growth restriction, hepatic mesenchymal hamartomas, and BWS). Additionally, this case supports the possibility of a common underlying etiology between hepatic cysts and PMD, as suggested by previous case reports. Due to the adverse outcomes related to fetuses with hepatic cysts and PMD, an MDT approach with early pediatric involvement is recommended. Additionally, while PlGF is a valuable marker of placental function in many contexts, this case, along with an unpublished one, challenges the prognostic reliability of PlGF levels in PMD and calls for further research.
